# Dyadic Invariance of the Positive Sexuality Scale in Chilean Heterosexual Couples

**DOI:** 10.3390/ijerph18031190

**Published:** 2021-01-29

**Authors:** Giulia Casu, Mónica Guzmán-González, Ricardo Espinoza-Tapia, Lusmenia Garrido-Rojas, Jaime Barrientos, Fabiola Gómez

**Affiliations:** 1 Department of Psychology, University of Bologna, 40127 Bologna, Italy; giulia.casu3@unibo.it; 2 School of Psychology, Universidad Católica del Norte, 1240000 Antofagasta, Chile; respinoza@ucn.cl; 3 Department of Psychology, Universidad Católica del Maule, 3460000 Talca, Chile; lgarrido@ucm.cl; 4 Faculty of Psychology, Universidad Alberto Hurtado, 8340575 Santiago, Chile; jbarrientos@uahurtado.cl; 5 School of Psychology, Pontificia Universidad Católica de Chile, 7691043 Santiago, Chile; fagomez3@uc.cl

**Keywords:** measurement invariance, positive sexuality, heterosexual couples, dyadic framework

## Abstract

Gender differences in sexuality-related dimensions have long been investigated in close relationship research. An important assumption when comparing values across gender in dyadic research is that both partners conceptualize the construct under investigation in the same way. Thus, issues of measurement invariance should be considered when working with dyadic data. The aim of the present study was to test the dyadic invariance of the Positive Sexuality Scale (PSS) to assess an individual’s sense of happiness and fulfillment with his/her sexual expression. The PSS was completed by 166 Chilean heterosexual couples, and measurement invariance was tested using confirmatory factor analysis within a dyadic framework. Configural, metric, scalar, and partial strict measurement invariance were supported for the PSS original one-factor model. No between-partner difference was found in the PSS latent factor mean. The functioning of the PSS and the meaning attributed to positive sexuality were the same for both partners. Hence, variations in the PSS levels between both partners in heterosexual couples can be interpreted as true mean differences rather than measurement artifacts.

## 1. Introduction

Gender differences in the subjective experience of inherently dyadic constructs, such as relationship quality and its multiple dimensions, have been extensively examined in close relationship research [1,2]. Gender differences have been especially reported for the relational construct of sexuality. For example, individual- and couple-based research indicates that men have a stronger sexual desire than women [3,4,5,6], which seems to lead to men taking the initiative in being physically intimate far more often than women [7,8]. Furthermore, it has been reported that, while men’s sexual desire is relatively stable across the life course, women’s sexual desire tends to change across the life span and the longer the relationship lasts [9,10]. However, some researchers suggest that these gender differences are highly gendered by cultural norms and social expectations, and more similarities than differences exist in sexual desire between men and women, e.g., [11]. A number of dyadic studies also indicate that men are more sexually satisfied than women, e.g., [5,6]; however, it is possible that men and women use a different set of criteria for evaluating their sexual satisfaction. Indeed, it has long been reported that motives for engaging in sex and feeling sexually satisfied are mainly focused on self-pleasure and sexual release for men and more relational and emotional for women [4,12].

In light of this, it is evident that, when comparing sexuality-related dimensions across partners in heterosexual couples, it is critical to first ascertain that both partners conceptualize and evaluate the construct under investigation in the same way. In other words, issues of measurement invariance should be carefully considered and formally tested by dyadic researchers before investigating gender differences [13], as only evidence of measurement invariance would allow meaningful comparisons of values and results in sexuality dimensions across gender [14]. Instead, dyadic invariance of relationship constructs is often simply assumed by researchers or tested without appropriately modeling the nonindependent nature of couple-level data [13]. A few exceptions exist: measurement invariance was tested using a dyadic analytical approach for measures of marital satisfaction [15,16] and spousal forgiveness [17], and findings generally supported the presence of weak (metric) invariance across dyad members [15,16,17]. However, to our knowledge, no sexuality-related construct has been tested for measurement invariance across partners in heterosexual relationships.

A recently proposed relational construct in the field of sexuality is positive sexuality. Within the sex-positive framework, positive sexuality refers to pleasure, expression, and contentment regarding sexual interests and behavior that strongly contribute to wellbeing [18]. Based on such a definition, a brief self-report measure was recently created and validated to assess female positive sexuality: the Positive Sexuality Scale (PSS) [19]. The PSS was developed using a focus group approach, and psychometric testing indicated that it is a valid and reliable one-factor tool of a woman’s sense of happiness and fulfillment with her unique sexuality and sexual expression that contributes to wellbeing. In light of its promising psychometric properties, a dyadic testing approach is worthy of being adopted to elucidate whether the PSS is invariant across male and female partners. This would enrich our understanding of whether the meaning attributed to positive sexuality is the same for both members of a heterosexual couple, also considering that the PSS was originally developed with and by women. Indeed, what it means to be happy and fulfilled with one’s unique sexuality may shift depending on whether one is a man or a woman.

Thus, the present study aimed to investigate whether invariance in the structure and nature of men’s and women’s positive sexuality can be reasonably assumed, by testing the PSS dyadic invariance across men and women involved in a committed heterosexual relationship. If invariance is achieved, then dyadic researchers will be able to meaningfully compare the levels of positive sexuality of both partners [14]. Being able to compare male and female partners in their levels of happiness and fulfillment with their sexual expression within the couple might contribute to furthering our understanding of gender differences in sexuality dimensions among heterosexual relationships.

## 2. Materials and Methods

### 2.1. Participants and Procedure

Participants were recruited through various strategies, such as posting a link to the online study on social networks (e.g., Twitter, Facebook) and snowball techniques, in which the researchers sent the study link to a list of e-mail contacts and asked each contact to forward the link to individuals from his/her environment (e.g., family members, friends, or acquaintances). Inclusion criteria were both partners being at least 18 years old, in a committed heterosexual relationship, and having been together for at least three months, with both partners willing to participate. Data collection was through the Survey Monkey platform. Before starting the survey, participants had to read and approve an informed consent. They were instructed to complete the questionnaire individually, and to not discuss the questions or answers with their partner. The study was approved by the University Ethics Board. Participation was voluntary.

Of the 170 couples who completed the questionnaire, 4 (1.7%) returned incomplete questionnaires. The final sample included 166 couples (166 men and 166 women). The mean age was 36.95 years for men (SD = 12.56; range 18–72 years) and 34.50 years for women (SD = 11.64; range 18–69 years). Two-thirds of the couples (68.67%, *n* = 104) were cohabiting or married. The mean relationship length was 10.58 years (SD = 10.32; range 5 months–46 years).

### 2.2. Measurement Instruments

Participants responded to a sociodemographic form (gender, age, marital status, length of relationship) and the PSS [19]. The PSS includes 5 items (e.g., “Sex with my partner is an exciting experience”) rated on a 7-point scale (1 = “strongly disagree” to 7 = “strongly agree”). Respondents were asked to think about their romantic relationship and to rate how much each item was representative of their sexual experience with their partner. The PSS was adapted to the Chilean context following a back-translation procedure. A validation study of the Chilean PSS is currently underway by the authors. Preliminary psychometric testing on a sample of 890 Chilean adults indicates a good fit of the original one-factor model, adequate internal consistency (Cronbach’s *α* > 0.70) and expected associations with relevant criterion variables (e.g., positive, large correlations with validated measures of relationship satisfaction and intimacy).

### 2.3. Data Analysis

Confirmatory factor analyses (CFAs) were first performed in men and women separately to ensure that the original PSS one-factor model could be used for subsequent measurement invariance testing. Dyadic measurement invariance was tested by comparing increasingly restrictive models that incrementally constrained additional model parameters to be equal across partners: configural (equal factor structure), metric (equal factor loadings), scalar (equal intercepts), and strict (equal residual variances) invariance. Differences in latent factor means between partners were then tested by setting the latent means as equal and comparing this model against the scalar invariance model. In the configural invariance model, the factor model for men and women was connected through a correlation between the latent factors, and error terms of parallel items were correlated between partners to account for nonindependence of observations at the factor and item levels. The maximum likelihood robust estimation was used. Model fit was evaluated using the Satorra–Bentler *χ*^2^ (S-B *χ*^2^), a comparative fit index (CFI) of ≥0.95, and a root-mean-square error of approximation (RMSEA) of ≤0.06 [20]. We based our evaluations of measurement invariance on both statistical and practical significance. From a statistical perspective, invariance was supported if nested models showed a nonsignificant decrease in model fit, as indicated by a nonsignificant S-B *χ*^2^ difference test (ΔS-B *χ*^2^). From a practical perspective, invariance was achieved if decrements in model fit across nested models were sufficiently small, as indicated by a decrease in CFI (ΔCFI) of <0.010 supplemented by an increase in RMSEA (ΔRMSEA) of <0.015 [21]. If full measurement invariance did not hold, partial measurement invariance was considered, which involves sequentially relaxing equality constraints on measurement parameters to determine which measurement parameters are noninvariant. Partial invariance is viable when at least two items per latent construct have invariant parameters [14]. As recently suggested for measurement invariance testing [22], the effects coding identification method was used, which allows for estimating the latent parameters in a nonarbitrary way by constraining the factor loadings to average 1 and the intercepts to sum up to 0. Sample size was established a priori so as to have at least 10 observations for each estimated parameter in the baseline configural invariance model [23]. Based on the literature, correlations of PSS scores with age and length of relationship were also computed. Correlation coefficients of 0.10 were considered small, 0.30 moderate, and 0.50 large [24]. Analyses were conducted using Mplus 7.2 (Muthén & Muthén, Los Angeles, CA, USA) for CFAs and IBM SPSS 25 (IBM, Armonk, NY, USA) for correlation analyses.

## 3. Results

The original one-factor model showed an adequate fit for both men (S-B*χ*^2^ (5) = 3.500, *p* = 0.624; CFI = 1.000; RMSEA < 0.001) and women (S-B*χ*^2^ (5) = 4.663, *p* = 0.458; CFI = 1.000; RMSEA < 0.001). The PSS one-factor model was thus subjected to testing for dyadic measurement invariance (Table 1).

The configural invariance model showed a good fit, indicating a similar factor structure of the PSS between partners. The correlation between men’s and women’s latent factors was 0.431 (*p* < 0.001). Correlations between parallel item residuals ranged from 0.026 to 0.229. Metric invariance was supported, as constraining all factor loadings as equal across partners did not worsen model fit at either a statistical or practical level. Thus, the strength of the association between each item and the latent factor was equivalent across partners, and the items were measuring the latent factor using the same metric scale across partners. The scalar invariance model also resulted in a nonsignificant worsening of fit from both statistical and practical points of view. Thus, partners with the same underlying level of the latent factor will have, on average, equivalent observed item scores. Full strict invariance was not supported, as constraining all residual variances to be equal across partners produced a statistically and practically significant loss of fit in relation to the scalar invariance model. Subsequent analyses revealed that the residual variance of item 3 (“Our intimate relationship is sexually stimulating”) was noninvariant, being higher for women (0.216) than for men (0.032) (ΔS-B *χ*^2^ (1) = 13.446, *p* < 0.001, ΔCFI = 0.023, ΔRMSEA = 0.038). When the equality constraint on the residual variance of this item was released, partial strict invariance was supported in both statistical and practical senses. Therefore, the explained variance for all PSS items except one was the same across both partners, indicating that the latent construct is measured equally across partners.

Given that scalar invariance was met, differences in latent factor means between partners were tested. The addition of equality constraints on the latent factor means yielded nonsignificant differences when compared to a model without these constraints. Thus, men (M = 5.409, SE = 0.656) and women (M = 6.636, SE = 0.877) had similar factor means.

Correlation analyses indicated that age was unrelated to PSS scores in men (*r* = −0.096, *p* = 0.224), whereas women’s age was negatively, weakly associated with PSS scores (*r* = −0.195, *p* = 0.012). Correlation of PSS with length of relationship was nonsignificant for men (*r* = −0.134, *p* = 0.086) but significant and negative for women, with a small effect size (*r* = −0.220, *p* = 0.004).

## 4. Discussion

Dyadic invariance is an important assumption to be tested before comparing values across individuals in a dyad, yet it has been largely overlooked in close relationship research [13]. Dyadic invariance testing is especially critical in the field of sexuality, as men and women might hold different conceptualizations of sexuality-related dimensions [4]. We tested the dyadic invariance of the Positive Sexuality Scale (PSS), a brief one-factor measure of positive sexuality developed within the sex-positive framework [19]. Our findings supported full configural, metric, and scalar invariance, and partial strict invariance, indicating that the PSS works in the same way for both members of Chilean heterosexual couples. Both male and female partners conceptualized positive sexuality as the subjective experience of positive feelings (happiness, amusement, enjoyment, pleasure) and the attribution of positive meaning and value (sense of fulfillment) to their own sexual experience within the relationship. Because full scalar invariance was achieved, partners can be meaningfully compared in the PSS latent factor means. Participating partners had the same underlying levels of positive sexuality. This result differs from what has been found in previous dyadic studies, with men reporting higher levels of sexual well-being [6,25]. It is worth noting that those studies were conducted in specific samples (i.e., couples in transition to parenthood or aging couples); hence, this finding deserves further exploration. The PSS also showed partial strict invariance, with invariant residual variances for 80% (4/5) of the PSS items, a proportion that is in line with standards for partial measurement invariance [14]. This implies that the PSS is equivalent in its measurement precision across partners and allows for meaningful interpretations of within-dyad comparisons also on observed means and covariance structures [26]. Noteworthy, strict invariance is rarely achieved in practice and considered to be excessively stringent [27,28]. Therefore, our findings further support our conclusions that the PSS can provide unbiased measurements of positive sexuality in Chilean partners.

We found that men’s positive sexuality was unrelated to age and relationship length, whereas for women, longer relationship duration and older age were associated with slightly lower positive sexuality. To our knowledge, no previous studies have related age and relationship length with positive sexuality. The only exception is the original validation study of the PSS [19]. That study reported slightly higher positive sexuality levels in younger women than in older ones, which is in line with our findings. Our results also seem coherent with research focused on different sexuality-related dimensions, which suggested a relative stability of sexual desire through men’s adult life [9,29] and a reduction in sexual desire over relationship duration in women but not in men [10,30]. It is noteworthy that the associations found in the current study were only weak, which seems to exclude a potential role of age and relationship length in the variations of PSS levels between partners of heterosexual couples. However, this issue is worthy of further investigation.

## 5. Conclusions

Besides further attesting the psychometric properties of the PSS, dyadic invariance testing enriched our scientific understanding of how positive sexuality is conceptualized by men and women in a committed heterosexual relationship. Because scalar and (partial) strict invariance were established, close relationship researchers and clinicians in Chile can confidently use the PSS to draw meaningful inferences about dyadic processes and interpret differences in the PSS between male and female partners as non-biased, conceptually meaningful, true mean differences rather than measurement artifacts. However, more research is needed to investigate the PSS dyadic invariance across couples with different characteristics (e.g., newlywed and/or same-sex couples, as well as couples seeking therapy).

## Figures and Tables

**Table 1:** Dyadic measurement invariance.

Level of Invariance	*df*	S-B*χ*^2^	SCF	Δ*df*	ΔS-B*χ*^2^	CFI	ΔCFI	RMSEA	ΔRMSEA
Configural	29	27.708	1.503	-	-	1.000	-	0.000	-
Metric	33	34.664	1.485	4	7.273 ^ns^	0.998	0.001	0.017	0.017
Scalar	37	39.814	1.430	4	4.144 ^ns^	0.997	0.001	0.021	0.004
Strict	42	72.683	1.815	5	16.067 *	0.971	0.026	0.066	0.045
Partial strict	41	46.445	1.769	4	5.142 ^ns^	0.995	0.002	0.028	0.007
Equal factor means	38	40.121	1.420	1	1.202 ^ns^	0.998	0.001	0.018	0.003

Note. *df* = degrees of freedom, S-B = Satorra-Bentler, SCF = scaling correction factor, Δ*df* = difference in *df*, ΔS-B*χ*^2^ = difference in S-B*χ*^2^, CFI = comparative fit index, ΔCFI = difference in CFI, RMSEA = root-mean-square error of approximation, ΔRMSEA = difference in RMSEA. ^ns^ *p* > 0.05. * *p* < 0.01.

## Data Availability

The data presented in this study are available on request from the corresponding author.
